# A Victory over Yellow Fever

**Published:** 1905-11-25

**Authors:** 


### A Victory Over Yellow Fever.

We had occasion to announce earlier in the year that the works on the projected canal across the Isthmus of Panama were to a great extent arrested by an outbreak of yellow fever. One or two cases were reported at the end of October last year, and a futile attempt was made to preserve secrecy with regard to them. But the rainy season set in, and the city was transformed into a morass, in every part of which fever-propagating mosquitoes could freely increase and multiply. Each month showed a lengthened roll of cases. Colonel Gorgas, the very competent chief of the health department, who had freed Havana from yellow fever in a few months, was almost helpless. He was hampered at every step by red tape, and seems to have been scarcely better off than an English medical officer of health to a municipality with a polluted water supply. This state of things continued until May of the present year, when President Roosevelt took the bit between his teeth and went ahead on his own responsibility. He compelled the old Canal Commissioners to resign, filled their places with new men, and sent Judge Magoon to Panama as Resident Governor of the Canal zone, with autocratic powers, which he promptly proceeded to exert. Space forbids us to enter into the details of his administration, but it is sufficient to say that he carried out an effective fumigation of every building in the city, ordered the immediate repairing of all window nettings, and made it known that anyone who tore holes in them or removed them would be severely punished; he divided the city into districts, to each of which a sanitary physician was appointed, with orders to make a daily house-to-house personal inspection of every inhabitant, so that every new case of fever should at once be notified. In the meantime all the water tanks and cisterns were closely screened against mosquitoes, all places where the insects could breed were destroyed or treated with oil or disinfectants, the work of providing a proper pipe-borne water supply was energetically pushed forward, so as to allow of all open tanks and cisterns being abolished, and the complete paving of all the streets with vitrified brick has been begun and is being rapidly carried through. The last case of yellow fever in Colon occurred on August 27, and the last case in Panama on September 14. In order to secure the early notification and effective protection from mosquitoes of any new case, the Government offers a reward of fifty dollars in gold for its detection and report. The mosquitoes must get their infection from fever patients in order to convey it to the healthy, and it is obvious that, if the isthmus can be kept free from fever, or if any fever patients that there may be can be protected from mosquitoes, during the period of lifetime of the insects, there will no longer be any possibility of a fresh outbreak. In a few weeks all the infected insects will be dead, and the isthmus will then be free from the disease, unless it should be reintroduced by importation from some other country.

The whole story is eminently worthy of record, if it were only for the practical lesson which it conveys. Local health authorities in this country always try to combat or to prevent disease by half measures, and they always fail. The half measures are nearly as costly as complete ones would be, and when they have been taken they are practically useless, because they leave without protection some point or points which it would be essential to complete security to guard. The epidemic comes in due time, and is probably almost as disastrous and as costly as it would have been if no half measures had been taken at all. The ordinary result is to produce a local conviction, not that the authorities are a set of incompetent blockheads, but that sanitary precautions are of little or no value. It is clear that, if Governor Magoon could have been given a free hand at Lincoln or at Basingstoke, the inhabitants of those places would not now be regretting the experiences through which they have passed.

